# Current and Future Trends in Strength and Conditioning for Female Athletes

**DOI:** 10.3390/ijerph19052687

**Published:** 2022-02-25

**Authors:** Anthony C. Santos, Tristan J. Turner, Dierdra K. Bycura

**Affiliations:** Department of Health Sciences, Northern Arizona University, Flagstaff, AZ 86011, USA; anthony.santos@nau.edu (A.C.S.); tjt348@nau.edu (T.J.T.)

**Keywords:** women athletes, training, functional movement, blood flow restriction, injury prevention, screening, technology

## Abstract

Participation by female athletes in competitive sport has increased dramatically since the inception of Title IX, although female athletes are represented significantly less than their male counterparts in strength and conditioning (S&C) literature. This is apparent when examining current identified trends in the field, such as implementation of blood flow restriction (BFR) training, functional assessments to predict injuries, or the ever-increasing use of technology in sports. The aim of this review is to examine three prevalent trends in contemporary S&C literature as they relate to female athletes in order to expose areas lacking in research. We conducted journal and database searches to progressively deepen our examination of available research, starting first with broad emerging themes within S&C, followed next by an inquiry into literature concerning S&C practices in females, ending finally with a review of emerging topics concerning female athletes. To this end, 534 articles were reviewed from PubMed, Academic Search Complete, Google Scholar, CINAHL, MEDLINE, and Web of Science. Results demonstrate the utility of implementing BFR, functional movement assessments, and various technologies among this population to expand representation of female athletes in S&C literature, improve athletic capabilities and performance, and decrease potential for injury over time.

## 1. Introduction

Incorporating female athletes into strength and conditioning (S&C) programs is relatively new due in part to the passage of Title IX in 1972. Title IX, a federal civil law, had a large impact on females in many different areas within educational institutions, although it arguably had the largest impact on sports participation [1]. Prior to the passage of Title IX, females did participate in sport, but their participation in S&C was far less than their male counterparts. Female athletes participating in sports prior to 1972 often engaged in more recreation-oriented activities, and few female athletes participated in a competitive environment. Unfortunately, cultural views during the early to mid-20th century did not favor elite performances among female athletes, and therefore female participation in S&C was viewed as unnecessary [2].

Title IX was certainly a catalyst for a large increase in the number of female athletes in sport at the professional and international levels [3]. The 1900 Olympic Games in Paris saw the first inclusion of female athletes with roughly 2.2% of females representing the total number of athletes at the Games (i.e., 22 females out of 997 total participants). In contrast, the 2012 Olympic Games in London showcased female athletes competing in every sport offered at the Games. The rise in female participation in the Olympic Games has grown immensely from its inception to a total of 45% of all participants in the 2016 Olympic Games in Rio de Janeiro [4].

Despite the growth in the participation of female athletes in sport, it seems that growth in S&C programs for female athletes moves at a slower pace. For example, 50% of high school coaches for male varsity student-athletes required their student-athletes to engage in strength training, compared to only 9% of coaches of female athletes [5]. To compound the issue further, there is a scarcity of published research focused on females. The average percentage of female participant inclusion per article within the sports and exercise medicine literature is between 35 and 37% [6]. It is possible that the historical lack of female participation in sport may contribute to this paucity of data, even as the rate of female participation has increased over the years. For example, blood flow restriction (BFR) as a training mechanism is a relatively new training method that is gaining popularity in S&C and rehabilitative settings. Inclusion of female participants in BFR studies is reported at 14% and 17% in studies of acute and chronic use, respectively [7], but only when searches are expanded to include recreationally active or untrained females outside of the 19–44-year-old age range (see Table below). Additionally, other trending areas of inquiry in S&C, such as functional assessments and wearable technology, also indicated a lack of studies that included female athletes as compared to their male counterparts.

The National Strength and Conditioning Association (NSCA) was founded in 1978 and is one of the first organizations to provide a certification for strength and conditioning coaches. In 1989, the NSCA released a position statement focusing on strength training for female athletes. Within the NSCA’s 1989 position statement, the organization stressed the importance of female role models in S&C as coaches [7]. A survey of 103 collegiate Division I head S&C coaches in 1990 found that 99% of head S&C coaches were male. Furthermore, the mean ratio of collegiate S&C staff was 2.6 males to 0.25 females at that time [8]. In 2016, 32% of all collegiate Division I (DI) S&C coaching positions were occupied by female S&C coaches [9]. However, DI male student-athletes reported being less comfortable with a female S&C coach, and would prefer working with a male S&C coach over a female S&C coach regardless of their qualifications [10]. Clearly, while there has been growth in the field of S&C for both female coaches and athletes, there are still attitudes towards females in S&C that may hinder their development. Addressing the social and structural determinants for females in S&C should help increase opportunities for both female athletes and coaches.

Given the increasing female participation in sports, there is a need to examine the implementation of current S&C practices in this population. The overarching goal of this narrative review is to identify the effects of emerging S&C training modalities on female athletes’ performance. More specifically, this narrative review synthesizes available literature regarding three identified prevalent trends in S&C (i.e., BFR training, functional assessments and screening, and the use of technology in sports) and the utilization of these trends with female athletes in order to guide future research for females participating in sports.

## 2. Methods

To prepare for the narrative review, a preliminary literature search for recent articles on training modalities employed among female athletes was conducted. Initially, we targeted two specific journals in which research of this nature is generally published in order to identify trends in the field of S&C in female athletes; namely, *Medicine & Science in Sports & Exercise* (MSSE) and the *Journal of Strength and Conditioning Research* (JSCR). Search criteria regarding publication date included articles that were published in the past eight years; this is the most distal time interval available on each journal’s Advanced Search Builder [11,12]. This broad initial search revealed approximately 2000 results. Three topics were then selected based on their prevalence in these peer-reviewed publications (see Table 1 for search result frequencies): blood flow restriction training, functional assessment and screening, and the use of technology in sports. After these topics were established, search terms for female subjects were added to further explore emerging S&C practices in females. Finally, a search term targeting practices with female athletes was added to specify this group as the focus of this review. Values presented in Table 1 are valid as of December 2021.

**Figure 1:**
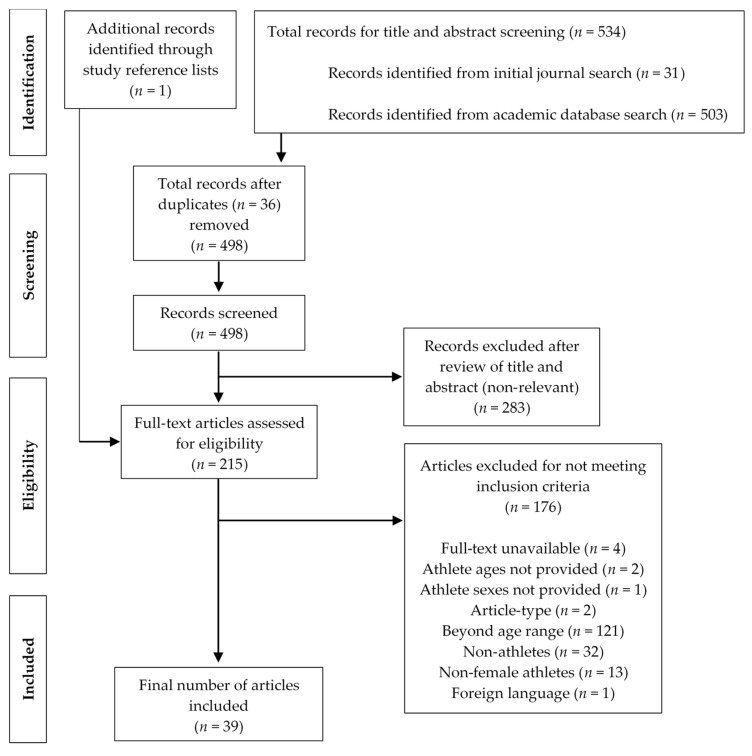
Flowchart of study selection.

A database search of the three trending S&C topics in female athletes was then conducted using PubMed, Google Scholar, EBSCOHost (Academic Search Complete, CINAHL Plus, and MEDLINE via Northern Arizona University’s Cline Library), and Web of Science. Search terms and filters utilized are described in Table 1. An emphasis was placed on gathering publications regarding adult female athletes, between the ages of 19 and 44 years, who were a part of a professional, national, major regional, or collegiate organization. Other inclusion criteria included full-text availability, peer-reviewed journal article (excluding review articles or dissertations, for instance), and English language (see Figure 1 for full criteria). Articles were excluded based on lacking focus on female athletes, inclusion of untrained or recreationally athletic participants, publication date earlier than 2011, and lack of relevance toward any of the trending topics chosen for review. Records were screened for duplicates and 534 publications were processed overall.

## 3. Blood Flow Restriction Training

BFR training is used in both S&C and rehabilitative settings. BFR aims to occlude venous return to the heart while allowing some arterial blood flow to the occluded area [13]. The reduction of arterial blood flow causes an ischemic state within the muscles. By further eliminating venous return to the heart, BFR forces swelling in the muscle distal to the cuff placement. The reduction of arterial blood flow, ischemic state, and occlusion of venous return increase the metabolic stress within the working muscles and lead to swelling of the muscle cells [14].

Therefore, occlusion pressure, or cuff pressure, is an important factor to consider when using BFR. Many research studies use a protocol of measuring arterial occlusion pressure (in mmHg) using an ultrasound Doppler probe. Cuff pressure is then inflated on the individual at 50–80% of the pressure required for complete arterial occlusion. It is important to recognize that arterial occlusion pressure values vary from person to person and thus arterial occlusion pressure should be tailored to the individual female athlete. Cuff placement is another important factor to consider when implementing BFR. Research protocols in the available literature typically place the cuff on the proximal portion of the limb, most commonly on the proximal thigh or arm. Proximal placement of the BFR cuff can affect distal muscle groups similarly to proximal muscle groups. BFR has been studied with both unilateral and bilateral cuff placement. Unilateral use of BFR has shown improvements on the contralateral limb that are similar to the improvements on the ipsilateral limb. This crossover effect suggests that results from the use of BFR may be systemic [15].

Various types of cuffs may be used in BFR, ranging from elastic bands to inflatable cuffs. Researchers have used a variety of different products in the implementation and study of BFR, though most available literature on females uses a system with an inflatable cuff that monitors and adjusts occlusion pressure during movements throughout the duration of the intervention session.

Although BFR was used as far back as 1983 by the general public, it has more recently been adopted in clinical settings. Yoshiaki Sato was a pioneer in the development of BFR training through the use of the KAATSU training method in Japan, first beginning in 1966 with self-experimentation [16]. Initial publications surrounding BFR have examined a predominantly male study population. The most widely cited reason for the exclusion of females in BFR studies is due to the menstrual cycle (MC) [17]. Few recent studies have sought to bridge the knowledge gap on the effects of BFR throughout the MC. One of the first studies to evaluate the hemodynamic effects of BFR training in untrained females during phases of the MC highlighted the need for future research examining the relationship between BFR and the MC [18].

The effects of BFR training on females are becoming increasingly better known, though use of female study populations remains relatively low. Studies focusing on females and BFR training have utilized objective measurements including the hemodynamic response to BFR training, aerobic effects (e.g., effects on VO_2max_), and anaerobic effects (e.g., effects on muscular power), muscular strength, and muscular hypertrophy. Furthermore, studies have also analyzed subjective measurements such as soreness and ratings of perceived exertion (RPE). Effects of BFR training have been analyzed using various levels of intensities, length of interventions, disease status, age, and more.

Hemodynamic responses to BFR have been measured using heart rate, cardiac output, and stroke volume. Low-load resistance training (RT) with BFR among older females has been shown to result in similar heart rate response and myocardial workload as high-load RT without BFR [19]. Among college-aged females, the use of BFR combined with low-load RT resulted in similar growth hormone and cortisol responses when compared to high-load RT without BFR [20]. Oxygen saturation rates (SpO_2_) are not significantly different when using BFR in low-load RT when compared to low-load and high-load RT without BFR [18]. The occlusion of blood that occurs is a key component of BFR, and may be responsible for the decreased amount of oxygen that is transported throughout the bloodstream, and is therefore available to muscles [21].

Table 2 provides a summary of selected BFR studies published between 2011 and 2021. Published research regarding the use of BFR in female athlete populations between 19 and 44 years of age is rare. It should be noted that, while the search strategies in this review followed strict guidelines for inclusion and exclusion of studies, criteria were relaxed here as the database searches only yielded three relevant articles. The table and discussion below are thus mostly populated by recreationally active and/or college-aged females, rather than dedicated female athletes.

The existing scientific literature examining BFR typically utilizes an exercise protocol of low-load RT with BFR, with intensities as low as 20% one-repetition maximum (1-RM) over 4–8 weeks [29]. However, some studies have examined BFR using aerobic training protocols. A 4-week randomized control trial (RCT) among healthy collegiate females used four different aerobic training protocols: (1) increasing pressure with increasing exercise, (2) increasing pressure with constant exercise, (3) constant pressure with constant exercise, and (4) constant pressure with increasing exercise. In all four groups, VO_2max_ and time to fatigue significantly improved. However, running economy decreased in all groups except for the increasing pressure and increasing exercise group. Results suggest that using aerobic training with BFR may lead to positive physiological changes in VO_2max_ and lengthen one’s exercise duration at the expense of running economy [22].

Other studies have examined BFR in RT utilizing higher 1-RM loads. Interestingly, Rawska et al. found that, when comparing total number of bench press repetitions (reps) performed at 80% 1-RM over five sets with ~80% full arterial occlusion of the upper arms, female RT athletes performed significantly more reps at a fast tempo (i.e., 2 s eccentric phase) with BFR (38.6 ± 4.0 reps) than without BFR (29.0 ± 1.7 reps) [27]. Similar results were also observed at a slow tempo (i.e., 6 s eccentric phase) with BFR (23.6 ± 0.5 reps) versus without BFR (19.3 ± 1.5 reps) [27]. Authors report that, while the use of BFR can improve the effectiveness of RT through augmented physiological and metabolic responses, the mechanical work generated by the strain of BFR cuffs can also affect one’s exercise capacity during BFR. Future studies should examine these mechanical effects when working with different means of blood flow restriction.

The safety of BFR is often discussed in terms of physiological changes associated with its use. The physiological changes that occur with the use of BFR, such as increased heart rate, blood pressure, and double product, support the recommendation that those using BFR should consult a medical professional prior to its use. A review of the literature surrounding BFR and many different safety concerns, such as venous thromboembolism, presence of reactive oxygen species, and muscle damage, highlights the need for future research to explore this area but also its safe use under the guidance of a medical professional [30]. BFR has been shown to be safe among healthy individuals. While further examination of these physiological changes among female athletes is necessary in the literature, it is also recommended that changes to athletes’ biomechanics be examined. A change in running economy while using BFR for aerobic exercise may predispose the female athlete to a risk of injury that otherwise may not be present without using BFR. Future studies should aim to utilize aerobic exercise with BFR and explore changes in running economy and gait with its use in order to provide more data and conclusions on the safety of BFR among female athletes.

Assessing an athlete’s soreness can also contribute to recovery. For instance, soreness levels after BFR exercise are similar to those after high-load training without the use of BFR [19]. In comparing low-intensity BFR training to high-intensity training among collegiate females, both subjective and objective assessments of soreness, such as rating soreness levels using a visual analog scale and blood lactate levels, were lower for participants using BFR [19,20]. RPE and lactate responses were significantly lower in the BFR group compared to the high-intensity training group as well, suggesting that the use of BFR among females will produce lesser or equal responses in soreness [20].

BFR can also benefit older adults through use of lower training load to achieve similar benefits as those provided by higher load training without the use of BFR. The use of BFR among healthy and active females, especially athletes, is quite limited. Thus, drawing results from an older population of females and applying it to younger and more active populations may not yield similar results. Nevertheless, a small sample of active females over a 4-week training program using BFR with low-load RT showed an increase in maximal voluntary contraction. Furthermore, training at loads as low as 25% of an individual’s 1-RM using BFR provides ample stimulus to improve plantar flexion 1-RM [31]. When combining BFR with whole body vibration training, vastus lateralis cross-sectional area (CSA) improved approximately 0.9 cm^2^ compared to a control group using only whole body vibration training, which improved 0.3 cm^2^, in a 10-week intervention [24]. Manimmanakorn et al. observed similar results, with CSA of the quadriceps and hamstrings increasing by 6.6 ± 4.5% in female netball athletes after a 5-week training period consisting of low-load RT (20% 1-RM) with KAATSU cuffs around the upper thighs [26]. Interestingly, BFR has not been shown to significantly increase flexibility through its use among females [23]. Therefore, low-load RT when combined with BFR can lead to greater muscle hypertrophy when compared to low loads without BFR. Furthermore, BFR with low-load RT shows similar results in hypertrophy improvements to those of high-load RT without the use of BFR.

While BFR training may be beneficial for certain populations at certain times, it is unlikely in S&C settings that BFR may be used in every training session, as different mesocycles may have different training and performance emphases with periods of detraining in between. Therefore, it is necessary to understand how a period of detraining might affect the female athlete after BFR training. In a 12-week detraining period following a 12-week training period, a loss of muscle size (i.e., muscle atrophy) and a decrease in muscular strength were found. However, the decreases in muscle size and strength were greater than pre-training measurements [28]. Weekly declines in muscular strength were also found in an RCT implementing a 6-week detraining period following a 16-week training period. The study supports the conclusion that, despite the decrease in muscular strength, the effects of BFR training on muscular strength are still greater than pre-training levels [25]. While decreases to muscle size and strength are expected after a period of detraining, these decreases are generally less severe following BFR. Future studies should aim to include a period of detraining following their training interventions to further understand the relationship between BFR and the effects of detraining.

Although BFR training has been around for many years, it remains an emerging trend. Despite its use, few studies exist that examine the impact of BFR in female athletes. Therefore, providing reasonable recommendations for the use of BFR among female athletes is difficult considering study populations typically use older or younger females who are only recreationally active. S&C is unique compared to other areas of fitness because its focus is on developing athletic performance. Future research should focus on the effects of BFR within S&C for females in order to address the literature gap regarding BFR and athletic performance among this population. Of the available scientific literature, BFR training has shown benefits for its use among females. The implementation of BFR into S&C programs may be limited due to the dearth of literature on its use, budget constraints, potential injury risks, and other factors. Therefore, S&C coaches may find the use of BFR beneficial when used in collaboration with sports medicine departments. By collaborating with sports medicine professionals, S&C coaches may be able to implement BFR training to aid in return to play for injured athletes who may have muscle atrophy, loss of strength, or decreased aerobic capacity of muscles.

## 4. Assessment, Screening, and Functional Training

Researchers and practitioners have sought to solidify functional movement practices by creating both screens and assessments. Screening allows practitioners to sample movement and determine if that movement is either limited and/or asymmetric, whereas an assessment further evaluates movement limitations or asymmetries using diagnostic criteria [32]. Screening and assessment are both purposeful and systematic in their approaches. Movement screening can be defined as a purposeful and systematic approach to identifying deficiencies in mobility and stability among asymptomatic individuals [32]. Providing a baseline of movement competency allows practitioners to form a foundation to design programs around. This is a necessary step in program design as it may help to decrease injuries caused by poor movement competency.

Several types of movement screens are currently being used among S&C coaches. In order to synthesize the literature on movement screens and assessments, this review will focus on the Functional Movement Screen (FMS), the Landing Error Scoring System (LESS), and the Y-Balance Test (YBT). Table 3 provides a summary of selected studies published between 2011 and 2021. The wide availability of different movement screening tools, such as the FMS, LESS, and YBT, aids in their implementation among S&C coaches. However, a limitation to the wide variety of options in movement screens is determining what movement screen is best.

The increase in participation in sports among collegiate female athletes is also coupled with an increased rate of injuries. For example, injuries to the lower extremity account for approximately 59% of all injuries for female NCAA basketball athletes [46]. Among those, anterior cruciate ligament (ACL) injuries are one of the most common and widely studied injuries among female athletes. Additionally, female collegiate athletes experience an increased incidence of sport-related injuries when compared to their male counterparts [34]. The high rate of lower body injury among female athletes has led to a surge in both research and clinical practice to prevent injury. Particular care should be taken by researchers and practitioners to identify and remain cognizant of the effects and interactions of the MC on non-contact injury risk in female athletes as well.

Among current trends in injury prevention is the use of movement screening. The purpose of a movement screen is to identify at-risk individuals, assist in program design through the use of corrective exercises to improve fundamental movement patterns, track development or improvement of movement patterns, and create a baseline measurement of fundamental movement with objective measurements [47]. By using a movement screen, S&C coaches can identify dysfunctional movement patterns and/or movement patterns that have not been developed properly. Dysfunctional movement patterns are inefficient and may be hiding movement compensations. Identifying movement dysfunctions allows the S&C coach to implement strategies to correct the dysfunction. Furthermore, correcting the movement dysfunction may lead to more efficient movements, which in turn may influence a positive change in biomechanics and ultimately help to reduce one’s risk of injury [44]. A common misconception of movement screening and assessments is its ability to predict athletic performance. Those who have scored higher in flexibility measures have been shown to score higher on the FMS, yet higher scores on the FMS or YBT were not significantly correlated with improved athletic performance [40,41,48].

### 4.1. The Functional Movement Screen

Among the available movement screens, three have been frequently used in the literature among female athletic populations: the FMS, the YBT, and the LESS. Among these three, the FMS is the most widely adopted screening tool for both researchers and clinicians. The FMS is composed of seven foundational movements (i.e., deep squat, hurdle step, inline lunge, shoulder mobility, active straight leg raise, trunk stability push-up, and rotary stability) pertaining to a range of mobility and stability competencies [43]. Each movement is performed barefoot over three trials, where the best rep is recorded and graded on a scale from zero to three, where a “0” indicates pain with the movement and “3” indicates proper form of the movement [35]. Therefore, the maximum composite score on the FMS is 21 points [49]. A composite score allows for a snapshot view of FMS overall; viewing only the composite score, however, does not allow the S&C coach to evaluate individual movement patterns that contributed to the overall score. Therefore, S&C coaches should aim to examine the scores of each movement pattern within the screen that contributed to the composite score. It has been suggested in the literature that a score of 14 or less is significantly correlated with risk of injury [40,50]. However, conflicting evidence exists in the literature as a score of 14 or more on the FMS has also been associated with an increased risk of injury. Therefore, the total score of the FMS should not be used as a sole predictor of injury [39,43]. A zero score on a movement in the FMS, indicating that the subject had pain with the given movement, may have implications as an early warning sign of injury [51]. Regardless, caution should be used when using a zero score on the FMS as a warning sign of injury, as data have also shown that pain during the FMS was not a predictor of injury [37].

The validity of the FMS as a predictor of injury also has varying results in the literature. Many studies appear to show that injury rates are higher among those who scored lower in the FMS when compared to those who did not sustain an injury during their sporting season [35,42]. Yet, many studies fail to reach statistical significance showing that a lower FMS score is associated with increased risk of future injury [34], while others state that the FMS is slightly better than 50/50 chance at accurately classifying those at increased risk for injury [36]. Analysis of the FMS pertaining to specific sports has also produced interesting results in the literature. Female combat sport athletes have scored higher, although not statistically significantly higher than football and basketball athletes [39]. Among female collegiate DI rowing athletes, the FMS was not a significant predictor of injury, and authors suggest that the FMS was not sport-specific enough to rowing (i.e., rowing utilizes multiplane spinal movements from a seated position, whereas the FMS does not) [35]. Rather than rely on composite scores, researchers instead suggest that individual component scores should be considered and used to implement athlete- and sport-specific strength and mobility programs to better correct movement dysfunctions, affect athletic performance, and reduce future injury risk [41].

The FMS also does not include a history section in its screening and thus does not record previous history of injury to a body part or region. Therefore, studies analyzing the FMS and injury prediction often do not include history of previous injury. Previous history of knee injury, or more generally, previous history of lower extremity injuries, has been reported as a risk factor for future injury to the area [38,52,53]. Female collegiate DI rowing athletes with a history of low back pain were six times more likely to suffer from low back pain during season compared to those who did not report a previous history of low back pain [35]. Therefore, S&C coaches should be aware of previous history of injury as it may show predictive signs of future injury.

Choosing to use any type of screen or assessment should come with a plan. Use of the data obtained from the screening or assessment and how that data might be implemented in S&C programs should be considered before implementing a movement screen or assessment. Female athletes will not benefit from the mere collection of movement data without the use of corrective exercises to combat individual movement dysfunctions. Among high school female student-athletes, researchers have found deficits in core strength. However, in a short prescribed corrective exercise program of 4 weeks, high school female student-athletes have shown improvements in their core strength [54]. Therefore, thoughtful use and application of a movement screen or assessment in programming for the female athlete may aid in addressing and correcting movement dysfunctions.

ACL injury is widely discussed among sports medicine and S&C coaches for female athletes. Female soccer athletes and female basketball athletes have been shown to have roughly a three times greater risk of ACL injury compared to male athletes [55]. This presents a concern for both sports medicine professionals and S&C coaches to address in the female athlete. A study among professional female basketball athletes in the pre-season found that the most common injuries during the season were knee related, accounting for a total of 40.2% of injuries. These included injuries to the ACL, medial collateral ligament (MCL), and lateral collateral ligaments (LCL). FMS displayed no significant difference in predicting injury between injured and non-injured professional female basketball athletes [39] or NCAA DI student-athletes [43]. However, injured professional female basketball players scored on average 1.3 times higher on the FMS compared to their non-injured counterparts [39]. Therefore, not looking solely at the composite score of the FMS and the addition of multiple screening tools may better help predict injury risk among female athletes.

### 4.2. The Landing Error Scoring System

Various other methods of gathering objective data on movement exist for female athletes, such as the LESS and YBT. Jumping mechanics are thought to play a role in non-contact ACL injuries, especially among females. Female athletes with a history of an ACL tear show an altered knee posture and loading compared to those who have not sustained a previous ACL injury. A previous history of ACL tear for female athletes is also associated with an increase in knee valgus upon landing [56]. The LESS is a validated and reliable tool for the assessment of landing mechanics after a jump [57]. The LESS is set up based on an athlete’s height; the athlete will jump forward from a 30 cm box towards the ground to an equivalent distance of half their height, then immediately perform a vertical jump upon landing. Viewing the athletes’ landing from the sagittal and frontal planes will allow the practitioner to examine the athletes’ landing characteristics in multiple planes to determine differences in movements such as angles of flexion/extension and abduction/adduction. Fatigue also plays an important factor in landing mechanics. For example, female athletes scored higher on the LESS after a period of intense lower body training [58]. The increase in knee valgus upon landing may be correlated to a lack of neuromuscular control of the knee [56], and future research should explore ACL injury prevention programming for female athletes focusing on neuromuscular control of the knee upon landing to avoid valgus collapse of the knee.

### 4.3. The Y-Balance Test

The YBT is a reliable method to objectively analyze dynamic balance [59]. Assessing dynamic balance using the YBT allows the practitioner to examine core and extremity function by quartering the body and placing it under body weight loads [60]. The YBT has both an upper quarter (YBT-UQ) and a lower quarter screen (YBT-LQ); for the purpose of this section, literature regarding the YBT-LQ will be emphasized. The YBT-LQ is similar to that of the Star Excursion Balance Test (SEBT) in that it tests single leg balance in multiple directions. However, it differs primarily in the number of directions tested. The three movements composed in the YBT-LQ are a single stance with contralateral limb reach in the anterior, posterior-medial, and posterior-lateral directions. Composite scores of the YBT-LQ and a modified version of the SEBT (so that the YBT tests the same three reaches) in healthy adolescent females did not differ, but scores did differ in the anterior reach direction [61]. Additionally, a small sample of 25 females who suffered an ACL injury demonstrated that there were no significant differences in the anterior reach direction when compared to non-ACL injured females [62]. Moreover, professional female basketball players improved their YBT-LQ composite, posterior-medial, and posterior-lateral scores after an 8-week intervention of neuromuscular training of bodyweight core and plyometric exercises. Therefore, S&C coaches may find it valuable to incorporate neuromuscular training into their warm-ups to aid in injury prevention by increasing joint awareness and postural control [33].

Performance measurements are common among S&C coaches to evaluate and track athletic-performance-based outcomes. Performance measurements have also been studied in the literature as a predictor of injury, such as a 10 m sprint, single leg three hop test, and 2k timed run. Within tactical populations, literature has shown that performance-based measurements have both higher sensitivity and specificity when compared to total FMS scores as a predictor of injury [51]. Performance-based measurements are often used at various points throughout the year for athletes. Performance measurements represent a cheap and easily implemented measurement tool for S&C coaches to use. Other examples of performance measures used by S&C coaches include the standing long jump, vertical jump, and pro-agility test. The pro-agility test significantly correlates with FMS composite scores in male high school athletes and YBT score in female high school athletes [63]. Currently, these measurement tools are primarily used to assess and track athletic performance. Limitations of the FMS include financial cost, requirement of a trained provider to implement it, and time associated with testing a respective group such as an entire sports team. Therefore, performance measurements such as 10 m sprint time, single leg three hop distance, 2 k run time, standing long jump, vertical jump, or the pro-agility test may provide a more effective predictor of injury. Future trends should aim to explore this relationship further. In addition, anthropometric measurements have been shown to have a more significant relationship with motor functions than the FMS [45].

In conclusion, a broad range of assessments and screenings such as the FMS, LESS, and YBT add to our understanding for better programming for sports performance. Injuries in athletics are often multifactorial and require holistic examination. Movement screening is just one piece of this holistic structure that can aid in the prediction and prevention of injury. Conflicting evidence exists for the relationship of injury in females in the FMS and YBT [39,62,63]. Therefore, S&C coaches should not rely solely on one movement screen. S&C coaches should utilize multiple methods to evaluate functional movement [63]. Future research on functional screening should aim to further analyze various different functional screening and assessment methods in their relationship with injury prevention. Scientific literature surrounding currently available movement screening and assessment tools should aim to incorporate females and more specifically female athletes as well.

## 5. Technology in Sports

As technology continues to advance, so does inclusion of technology in S&C. Generally, the increased use of technology in sports often incorporates various aspects of performance tracking, recovery tracking, and integration of data into larger programming strategies. Most of these measurements deal with internal physiological responses to training (e.g., HRV, oxygen saturation, response times, etc.) as well as larger sport-related movements during practice or competition (e.g., change of direction, acceleration or deceleration, quantifying training and player load, etc.). Researchers should understand how technology is being integrated into larger S&C practices in order to remain abreast of current and emerging trends. The inclusion of technology into S&C can provide numerous objective data points with which coaches and researchers can design, monitor, and assess training programs to better promote physiological adaptations, reduce injury risk, and advance return-to-play protocols following rehabilitation [64,65]. However, the inclusion of females and more specifically female athletes is severely underrepresented in the literature.

Table 4 provides a summary of the selected studies published between 2011 and 2021.

### 5.1. Velocity-Based Training

Programming is an integral part in preparing the female athlete for athletic performance. Proper set and repetition schemes with appropriate rest intervals can play a factor in the female athlete’s performance. Additionally, training loads should be prescribed based on training goals and training phase. One repetition maximum testing is widely used among S&C professionals to prescribe training loads as a percentage of 1-RM (%1-RM), or relative load. Movement velocity may offer an additional method of measuring load percentages for programming in female athletes. Weakley and colleagues refer to velocity-based training as “a method that uses velocity to inform or enhance training practice” [79] (p. 31). Load-velocity relationships among females have been proven to be accurate both in stronger and weaker females [80]. This finding provides evidence to support the use of barbell velocity as an alternative measure to assessing 1-RM and using %1-RM in programming regardless of the strength of the female athlete, as suggested by Torrejón et al. [80]. Interestingly, female athletes have shown higher barbell velocity when performing heavy loads on bench press compared to males. However, females showed lower barbell velocity when performing lighter loads on bench press compared to males [81].

The literature on the use of velocity-based training in S&C is lacking in female athlete inclusion [82]. It has been found that among males, the use of barbell velocity training can have a positive effect on training. Training at higher maximal velocities leads to greater gains in strength compared to lower velocities [83]. Barbell velocity-based training is becoming popular within S&C for a variety of reasons, one of which is the feasibility of its application. Products that measure barbell velocity are typically used in research to detect changes in speed. For example, Bozzini and colleagues utilized a TENDO unit attached to a 20 kg barbell to measure jump velocity among female NCAA DI beach volleyball players [64]. Similarly, McKeown et al. used a linear position transducer attached to either a wooden dowel or 15 kg barbell to assess peak movement velocity, mean concentric power, and maximum jump height during multiple countermovement jumps among female netball players [72]. Measurements of force, which are important in determining power, can be measured using barbell velocity measurements through change barbell speed, or by using force plates to assess ground reaction forces, contact time, and flight time, and to calculate the reactive strength index [72,84]. Linear velocity transducers come in many different styles, including smartphone applications with Bluetooth connectivity, making the consumer market rather versatile for their implementation.

Maintaining velocity throughout the training session in strength-based phases is important as training at higher velocities rather than lower velocities can lead to greater strength gains [83]. The greatest difference in average training velocity using the bench press was observed in the last repetitions of the set [81], suggesting that velocity may decrease as repetitions increase for the bench press. Furthermore, the addition of longer intra-set rest periods may aid in recovery between sets to combat this fatigue, although future research is needed to further support this, especially among females.

### 5.2. Wearable Technology—Motion Analysis Systems

Global positioning systems (GPS) have been increasingly studied in recent years for player tracking [64,65,67,85]. This tracking allows for movement analysis on the field or court, which can be useful for female athletes who engage in sports that require running as the primary means of locomotion, along with change of direction. One challenge is that current technologies often limit the range of data collection based on wireless range capability, although quality data on athlete performance can still be tracked. For instance, Benjamin et al. report device biases of only 1.99 ± 1.81% for 400 m running, 1.26 ± 1.04% for 20 m linear running, and 1.80 ± 1.93% for peak speed for one wearable GPS tracker [67]. Literature surrounding GPS systems in female sports includes topics such as distance traveled, training load, player load by position (among NCAA DI female soccer athletes), exercise energy expenditure, maximal speed, acceleration/deceleration, and duration of high-intensity running [64,65,85,86]. Among elite female field hockey players, it has been shown that different positions have varying physiological demands associated with frequency of high-intensity efforts and total distance covered. This has been shown in female volleyball [78] and soccer [65] athletes as well. In order to meet specific demands of each position, programming in S&C should be individualized to the female athlete to match their specific position demands [87].

Recovery is also a crucial component for female athletes to maintain athletic performance. Suggestions in the literature have pointed to using distance tracking as a way to measure fatigue during matches, as this may influence different strategies for substitutions in team sports and may aid in injury prevention for injuries related to fatigue [86]. While S&C coaches should remember to train a baseline or foundational movement pattern specific to the individual, research among South African semi-elite female soccer players suggests that training for specific positions within sport may increase athletic performance directly related to the female athlete’s role in their sport [76]. However, caution should be taken when interpreting and applying these results to various age populations of female athletes. Sport specialization may negatively affect physical literacy but may also have a negative financial impact on one’s family due to an increased potential for overuse injuries and healthcare costs [88].

GPS devices represent one of many options in the wearable technology field in sport and exercise. Their implementation is as simple as attaching a GPS device to the female athlete. The consumer market of wearable technology revolves, in part, around simplicity of use. This simplicity has driven the market for wearable technology upwards and has expanded its reach. Other examples of wearable technologies within the literature among female athletes include heart rate (HR) monitors to measure variables such as training load or HRV and corroborate RPE with physical performance [64,73,85]. Non-GPS movement-based wearables are also available that can track accelerations, decelerations, jumps, and changes in direction [74,78]. Such devices provide greater reliability when used indoors and rely on gyroscopes, magnetometers, and accelerometers rather than communication between multiple GPS satellites. These microsensor technologies have been used to longitudinally determine player loads during competition by position and game outcome among NCAA DI female basketball players [74], as well as player load, high-impact player load, explosive efforts, and jumps among NCAA DI female volleyball players [78]. The use of wearable technology for exercise to meet the American College of Sports Medicine’s (ACSM) weekly exercise recommendations has also been shown to increase adherence to exercise programs [89]. Many of these devices in the literature are wrist-worn devices, which may aid in adherence when using these wearables due to their ease of use. In these ways, wearable devices can provide normative data and/or real-time monitoring to observe training and competition session loads and track recovery, and ultimately enhance athletic performance [73,74,78].

### 5.3. Sleep Monitoring

Sleep has widely been known to influence athletic performance. Wearable and sheet-type devices have allowed for monitoring of sleep habits, which may provide more data to support sleep patterns and their influence on training and performance [71]. Heart rate variability (HRV), or the time between heartbeats, has been studied as a means of assessing cardiac health and the state of one’s autonomic nervous system (ANS), which aids in regulating cardiac activity [77,90]. Female cyclists competing in the Tour de France showed that HRV indices increased over days correlated with increased load within the race. Interestingly, these female cyclists also showed that one week of recovery post-race was necessary to return heart-rate variability to pre-race values [66]. Among high-level female soccer players, disrupted individual sleep patterns were identified in that HRV differed among individuals and training loads. In this case, as well as in female intercollegiate middle-distance runners, higher workloads were associated with more sleep disruptions [68,71]. In terms of changing or improving sleep patterns, a study among female African-American college students revealed that stand-alone wearable technologies for sleep showed no effect in improving sleep [91]. This suggests that wearable technologies for sleep may be best implemented to track sleep data, but the technology may not be the best method for changing sleep habits.

In another study among high-level female soccer athletes, it was suggested that tracking both subjective and objective sleep measurements, such as perceived sleep quality and waking HRV measures, respectively, may allow coaches to make informed decisions on load management or fatigue in female athletes [70]. Such observations of load management and fatigue were trialed by Tian et al., who utilized HRV measurements at night before sleep to identify functional overreaching and nonfunctional overreaching states among elite female wrestlers [77]. While some measures of HRV can be costly to implement, some research is beginning to emerge using smartphone applications for recording HRV and may be useful in monitoring the effects from training for female athletes in a team sport [69]. Future research should aim to explore the benefits of implementing sleep data from wearable technology in adjusting sleep habits for female athletes who do not receive 7–8 h of undisturbed sleep.

In general, technologies in sports used to monitor various elements of training and recovery, such as training velocity, motion analysis, and sleep quality, have gathered a considerable amount of attention in the sport and exercise science fields. We acknowledge the obvious breadth of this section and only provide an extremely brief overview of contemporary uses of technology in S&C practices with female athletes. Understandably, this section could be expanded into its own review, or several reviews, and the larger S&C literature would be greatly benefited by additional in-depth reviews of subtopics presented here. The future of technology in sport is likely to continue on an upward trend with the advancement of technology. As the market for wearable devices continues to increase, the inclusion of female athletes into the scientific literature on wearable technology should grow similarly.

## 6. Conclusions

The growth in female participation in sport has increased immensely and it is time for the scientific literature to catch up with the upward trend in female participation. Performance in elite levels of competition was not as widely available or part of programming for the female athlete prior to Title IX. The passage of Title IX in 1972 created a pivotal shift towards the inclusion of females in both sport and S&C. The increase in participation is evident in the inclusion of females in competitive sports from youth levels to the Olympic and Paralympic Games. The 1989 position statement from the NSCA summarized their recommendations on training the female athlete by stating that “males and females should train for strength in the same basic way, employing similar methodologies, programs, and types of exercises” (p. 30) [92]. Thirty years after the NSCA’s position statement, in 2018, a narrative review concluded that the NSCA’s recommendation from 1989 still holds true [93].

BFR training may have implications for female athletes, especially when the training goals involve return-to-play after injury. The addition of BFR training in female athletes after injury may aid in their ability to combat muscle atrophy by promoting muscle growth under lighter loads. Functional screening is an important aspect of the S&C coaches’ program as well, and S&C coaches should use a broad approach to assess, screen, and analyze movement holistically, rather than rely on one single movement assessment. Additionally, the incorporation of neuromuscular training for the lower extremity may aid in injury prevention for the female athlete through improved joint awareness and postural control. Sports technology may also continue to grow in popularity and utility among female athletes. Sports technology may aid in tracking and monitoring specific training and physiologic data such as barbell velocity, sleep quality, and HRV. Use of technology in sports may also aid in developing or modifying performance measures to better characterize athletic abilities in female athletes beyond previously used tests [75]. This data may also aid the S&C coach in implementing and/or adjusting programs to improve the female athlete’s performance and recovery. The common thread among all three current topics presented in this review is a lack of inclusion of females in the sport and exercise science literature. As these current trends continue to grow and as new trends begin to emerge within the sports and exercise science community, the inclusion of female athletes in the scientific literature should be emphasized.

Narrative reviews are not without their limitations. This review examined the literature through a narrative rather than a strictly systematic lens. Systematic reviews offer a broad summary of the literature using explicit and reproducible methods to comprehensively search, critique, and synthesize research on a specific topic or issue [94]. Whereas systematic reviews may also include a meta-analysis of statistical data, a narrative review lacks this analysis of data, but still offers an opportunity for synthesis of available literature. Furthermore, a second limitation of this study is that the inclusion of females in the literature is low. Therefore, the available literature to be synthesized is of lower quantity compared to that of men. The lower quantity of literature available also serves to highlight the need for the inclusion of females as research participants. The literature surrounding BFR and athletic performance is inherently low in quantity as well. Synthesizing recommendations for athletic performance improvements using BFR training from study populations that fail to include athletic females provides a disservice to this population. Future research should aim to further analyze the gap in BFR training for female athletes in improving athletic performance. Additionally, functional assessment must first start with either a screen or assessment of functional movement or capacity of the individual. Future research on functional screening should aim to evaluate the conjunction of multiple different screening and assessment tools in their ability to predict injury in female athletes. Finally, limitations also exist in the available literature on the role of technology in sports for female athletes. Future research should work to increase understanding of how various technologies could benefit female athletes in S&C and sports.

## Figures and Tables

**Table 1:** Initial literature and database search strategies.

Literature Search Strategies ^a^			
Journal		Search Terms	Results (*n*)
MSSE		Blood flow restriction training	184
		(female OR women) AND blood flow restriction training	70
		(female OR women) AND athlete* AND blood flow restriction training	13
		Functional assessment AND screening	188
		(female OR women) AND functional assessment AND screening	126
		(female OR women) AND athlete* AND functional assessment AND screening	63
		Technology in Sports	470
		(female OR women) AND technology in sports	378
		(female OR women) AND athlete* AND technology in sports	153
JSCR		Blood flow restriction training	93
		(female OR women) AND blood flow restriction training	44
		(female OR women) AND athlete* AND blood flow restriction training	33
		Functional assessment AND screening	168
		(female OR women) AND functional assessment AND screening	113
		(female OR women) AND athlete* AND functional assessment AND screening	87
		Technology in Sports	580
		(female OR women) AND technology in sports	323
		(female OR women) AND athlete* AND technology in sports	285
**Academic Database Search Strategies**			
**Database**	**Search Terms ^b^**	**Filters Applied**	
PubMed	(“female” OR women) AND athlete* AND (“strength and conditioning”) AND (“blood flow restriction training” OR functional (assessment OR screening) OR technology OR sports)	Free full text, Full text, Humans, English, Adult: 19–44 years, Female, from 2011–2021.	
Google Scholar		Custom date range: 2011–2021. Sort by relevance. Articles: any type. Include citations.	
EBSCOHost ^c^		Full Text; Scholarly (Peer Reviewed) Journals; Published Date: 1 January 2011–31 December 2021; Language: English; English Language; Human; Sex: Female; Age Groups: Adult: 19–44 years; English Language; Human; Sex: Female; Age Related: Adult: 19–44 years. Expanders—Apply related words; Apply equivalent subjects. Search modes—Boolean/Phrase.	
Web of Science		Timespan: 1 January 2011 to 31 December 2021 (Index Date). Languages: English.	

Note. JSCR: *Journal of Strength and Conditioning Research*; MSSE: *Medicine & Science in Sport & Exercise*. ^a^ “Articles” were selected for Content Type while “Last 8 Years” was selected for Publication Date for both journals. ^b^ Search terms were applied to all fields during database searches. ^c^ Academic Search Complete, CINAHL Plus, and MEDLINE were accessed via EBSCOHost at Northern Arizona University.

**Table 2:** Summary of blood flow restriction studies among female athletes (2011–2021).

Study [Reference]	Participants	Exercise Protocol	Cuff Pressure	Conclusions
Amani-Shalamzari et al., 2019 [22]	32 active collegiate females aged 18–30 years.	Exercise: 12 sessions of 2 min treadmill run w/BFR. Four groups with varying pressures (increasing or constant) and exercise intensity (increasing or constant).	Pressure varied by group. Min. pressure: 160 mmHg. Max. pressure: 240 mmHg.	VO_2max_ and anaerobic parameters increased in all groups. Constant complete pressure of 240 mmHg with increasing exercise intensity showed the greatest gain in muscular strength.
Araujo et al., 2017 [23]	29 untrained females aged 19–39 years.	Exercises: biceps curl, knee extension. LL_BFR_: 1 × 30, 3 × 15 20% 1-RM HI: 4 × 8 80% 1-RM Control-ADL’s Duration-8 training sessions × 2/week	80% of complete arterial occlusion at rest.	No significant increase in flexibility for all groups. HI and LL_BFR_ had significant increase in maximal dynamic strength in different phases of the MC.
Centner et al., 2020 [24]	50 recreationally active females aged 18–40 years.	Exercises: squat, isometric squat at 120°. Plantar flexion, isometric plantar flexion at end range. Vibration frequency: 30 Hz Amplitude: ramping between 2–4 mm Dynamic: 3 × 15. Isometric: 3 × 45 s. 10 weeks × 3/week	50% arterial occlusion pressure.	Whole-body vibration + BFR increased vastus lateralis CSA from 17.4 ± 2.2 cm^2^ to 18.3 ± 2.3 cm^2^. Comparatively, whole body vibration training increased vastus lateralis CSA from 19.2 ± 3.0 cm^2^ to 19.5 ± 2.6 cm^2^.
Kim et al., 2014 [20]	13 recreationally active females aged 18–25 years.	Exercises: 2 sessions of isotonic knee extension and leg press. LL_BFR_: 1 × 30, 2 × 15 20% 1-RM HL-3 × 10, 80% 1-RM	200 mmHg.	LL_BFR_ and HI had similar GH and cortisol responses. HI had higher RPE and lactate responses than LL_BFR_.
Letieri et al., 2018 [25]	56 females aged 68.8 ± 5.09 years.	Exercises: squat, leg press, knee extension, and leg curl. LL_BFR_ w/high pressure and LL_BFR_ w/low pressure: 3–4 × 15 20–30% 1-RM HI-3–4 × 6–8 70–80% 1-RM Control: ADL’s 16 weeks, ×3/week 6-weeks detraining	LL_BFR_ w/high pressure- 185.75 ± 5.45 mmHg. LL_BFR_ w/low pressure-105.45 ± 6.5 mmHg.	LL_BFR_ w/both high and low pressures showed similar increases in strength as the HI group.
Manimmanakorn et al., 2013 [26]	30 female netball athletes, mean age 20.2 ± 3.3 years.	5 weeks of LLRE (20% 1RM) for knee flexor and extensor muscles with: (1) BFR occlusion around the upper thighs, (2) normobaric hypoxic gas, or (3) no additional stimulus. Freq.: 3 d·wk^−1^ of 3 sets of knee extensions to failure, followed by 3 sets of knee flexions to failure, with 30 s rest between sets and 2 min rest between exercises.	~230 mmHg.	The exercise protocol with either BFR or hypoxia increased muscular strength, endurance, and CSA compared to control training. BFR also improved sport-specific fitness test outcomes over hypoxic and control training.
Neto et al., 2017 [18]	30 untrained females aged 21.7 ± 3.4 years.	Exercises: biceps curl and knee extension. LL: 1 × 30, 3 × 15 20% 1-RM LL_BFR_: 1 × 30, 3 × 15 20% 1-RM HL: 4 × 8 80% 1-RM 26 days	80% of total arterial occlusion pressure.	All groups increased SBP, HR and DP but did not increase SpO_2_. Greatest increase in HR and DP in the luteal phase.
Rawska et al., 2019 [27]	4 experienced female RT athletes, mean age 27.3 ± 2.2 years.	Four sessions of: 5 sets of AMRAP bench press at 80% 1 RM to a fast or slow tempo with 3 min rest between sets.	~80% full arterial occlusion.	Both BFR and tempo produced significant effects on maximum reps per set. Fast tempo with BFR resulted in more reps than fast tempo without BFR, as well as slow tempo with BFR versus without.
Scott et al., 2018 [19]	15 females aged 63–75 years.	Exercises: Leg press and leg extension. LL: 1 × 20, 2 × 15 20% 1-RM LL_BFR_: 1 × 20, 2 × 15 20% 1-RM HL: 3 × 10, 70% 1-RM 3 sessions	50% arterial occlusion pressure.	LL_BFR_: greater SBP, DBP and MAP than HL. LL_BFR_ and HL have similar HR responses and myocardial workload. Soreness levels were similar in all groups.
Yasuda et al., 2015 [28]	14 Japanese females aged 61–85 years.	Exercises: arm curl, triceps press down with thin yellow band. LL_BFR_ & LL group 1 × 30, 3 × 15 Duration: 12 weeks, ×2/week. 12-week detraining.	Started at 120 mmHg and progressed to a max of 270 mmHg.	Magnitude of change of muscle CSA between pre- and post-exercise was always larger in the LL_BFR_ group. Twelve-week detraining reduced muscular strength and size, but they remained higher than pre-training levels.

Note. ADL: activities of daily living; AMRAP: as many repetitions as possible; BFR: blood flow restriction; CSA: cross-sectional area; DBP: diastolic blood pressure; DP: double product; Freq.: frequency; HI: high intensity; HL: high load; HR: heart rate; LL: low load; LL_BFR_: low load with blood flow restriction; LLRE: low-load resistance exercise; MAP: mean arterial pressure; MC: menstrual cycle; mmHg: millimeter of mercury; MVC: maximal voluntary contraction; RT: resistance training; SBP: systolic blood pressure; SpO_2_: oxygen saturation; w/: with; w/o: without; 1-RM: 1-repetition maximum.

**Table 3:** Summary of functional assessments and screening studies among female athletes (2011–2021).

Study [Reference]	Participants	Assessment/Screening Tools Used	Conclusion
Benis et al., 2016 [33]	28 elite female Italian national league basketball players aged 20 ± 2 years.	YBT	Eight-week neuromuscular training program (body weight core stability and plyometric exercises) improved composite YBT scores for both lower limbs from baseline in EXP group as compared to CON group. Improvements in posterolateral and posteromedial directions were seen in EXP group, but not in anterior reach.
Brumitt et al., 2018 [34]	106 NCAA DIII female soccer, volleyball, XC, basketball, lacrosse, tennis, softball, and track athletes, mean age 19.1 ± 1.1 years.	SLJ SLH LEFT	Suboptimal scores on each test were associated with significantly increased risk for initial and total time-loss LQ injury, particularly at the thigh or knee. At-risk athletes with a history of LQ sports injuries and less active off-season training habits had an 18-fold increased risk of a time-loss thigh or knee injury during the season.
Clay et al., 2016 [35]	37 NCAA DI Collegiate female rowers, mean age 19.4 ± 1.2 years.	FMS	Previous history of LBP resulted in being 6 times more likely to experience LBP during the season. Greater rowing experience (years) was associated with higher reports of LBP. FMS was not statistically significant in predicting time loss injury in female collegiate rowers.
Dorrel et al., 2018 [36]	257 NCAA DII female (*n* = 81) and male (*n* = 176) collegiate athletes, ages between 18 and 24 years.	FMS	A cutoff score of ≤15 was associated with relative risk of 1.25, 1.25, and 1.45 for musculoskeletal, overall, and severe injuries in this sample, respectively. Due to AUC scores of 0.54, 0.56, and 0.53 for musculoskeletal, overall, and severe injuries, respectively, authors regard the FMS as slightly better than chance at predicting injuries among this group.
Landis et al., 2018 [37]	187 female NAIA varsity-level soccer, basketball, and volleyball players, mean age 19.5 ± 1.21 years.	FMS Drop-jump landing	Non-contact LE injured participants (*n* = 17) scored significantly lower on the FMS (14 ± 3.46 and 15.35 ± 2.58). Previous ACL injury demonstrated lower FMS scores (13.84 ± 3.611) compared to non-injured participants (15.30 ± 2.732) and significantly predicted future LE injury. Poorer drop-jump landing mechanics were not significantly associated with injury, though trends existed.
Ness et al., 2016 [38]	17 NCAA DI female soccer athletes, mean age 18.8 ± 0.9 years.	SEBT Isometric hip strength	Following 8 weeks of offseason training, SEBT composite reach distance improved in both dominant and non-dominant limbs. Dominant hip external rotation strength gains also appear to be associated with improved lower extremity dynamic balance.
Šiupšinskas et al., 2019 [39]	169 professional female basketball players of the XWBL league, mean age 23.1 ± 5.7 years.	YBT-LQ FMS LESS	Injured players (*n* = 92) scored 1.3 points lower on the FMS (14.1) than non-injured (*n* = 77) players (15.4) and 1 point higher on the LESS (8) than non-injured players (7). Group differences for YBT-LQ scores were not statistically significant.
Sprague et al., 2014 [40]	57 NCAA DII female (*n* = 37) and male (*n* = 20) volleyball and soccer athletes, mean age 19.7 ± 1.3 years.	FMS	FMS composite scores did not differ between pre- and post-season, although all teams trended toward improvements. All teams reduced total number of asymmetries between measurements as well. In terms of individual movements, all athletes improved on the deep squat and in-line lunge, while worsening on the active straight leg raise and rotary stability movement.
Stapleton et al., 2021 [41]	38 NCAA DI male (*n* = 23) baseball and female (*n* = 15) softball athletes, mean age 20.0 ± 1.38 years.	FMS YBT-UQ YBT-LQ Athletic performance (vertical jump, pro-agility, rotational medicine ball throw)	In female softball athletes, significant negative correlations were found between composite FMS and RMTR; between RMTR and FMS in-line lunge, YBT-LQ anterior reach, and YBT-LQ posterolateral reach; and between pro-agility and YBT-LQ posterolateral reach and YBT-UQ superolateral reach. Vertical jump was significantly positively correlated with YBT-LQ posterolateral reach and YBT-UQ superolateral reach. Overall, composite scores of FMS, YBT-LQ, and YBT-UQ did not significantly predict total performance. Individual components (active straight leg raise and hurdle step) significantly predicted total performance.
Walbright et al., 2017 [42]	35 NCAA DI collegiate female basketball (*n* = 17) and volleyball (*n* = 18) players.	YBT FMS SLH	FMS, YBT, and SLHT did not show a relationship between composite score and lost time LQ injury. The tests were not predictive of LQ injury occurrence.
Warren et al., 2015 [43]	167 NCAA DI female (*n* = 78) and male (*n* = 89) collegiate athletes, mean age 20.3 ± 1.5 years.	FMS	No association between FMS composite score and non-contact injury was found within this sample. Authors also found no association between FMS movement pattern asymmetry and injury. In comparison to other studies, the FMS might be better suited at predicting injury in contact or traumatic injuries.
Warren et al., 2020 [44]	68 NCAA DI female basketball, soccer, and volleyball athletes, mean age 19.1 ± 1.1 years.	SLH THT XCT Isometric hip strength	THT score significantly predicted non-contact injury risk in this group. Athletes in the weakest tertile for hip external rotation strength were at increased odds of injury compared to the strongest tertile as well.
Zibaie et al., 2019 [45]	58 Iranian female athletes, mean age 21.11 ± 7.71 years.	FMS Core proprioception (using gyroscope) Anthropometric data	Correlations between FMS composite scores and core proprioception and anthropometric dimensions were not statistically significant.

Note. AAA: Athletic Ability Assessment; ACL: anterior cruciate ligament; AUC: area under the curve; CON: control; DI: division one; DII: division two; DIII: division three; EXP: experimental; FMS: Functional Movement Screen; FPT: Functional Performance Test; LBP: low back pain; LE: lower extremity; LEFT: Lower Extremity Functional Test; LESS: Landing Error Scoring System; LQ: lower quarter; NAIA: National Association of Intercollegiate Athletics; NCAA: National Collegiate Athletic Association; RMTR: rotational medicine ball thrown to the right; SEBT: Star Excursion Balance Test; SLH: Single Leg Hop for Distance Test; SLJ: Standing Long Jump Test; THT: Triple Hop Test for Distance; XHT: Crossover Hop Test for Distance; XWBL: X Women’s Basketball League; XC: cross-country; YBT: Y-Balance Test; YBT-LQ: Y-Balance Test, Lower Quarter Screen; YBT-UQ: Y-Balance Test, Upper Quarter Screen.

**Table 4:** Summary of technology in sports studies among female athletes (2011–2021).

Study [Reference]	Participants	Measurement/Technology	Conclusion
Barrero et al., 2019 [66]	10 European regional- or national-level female cyclists, mean age 31.7 ± 4.7 years.	HRV (1000 Hz HR monitor)	Higher daily workload from intense exercise correlated to higher supine HR after a recovery night. One-week rest from intense exercise was enough to restore baseline HRV values after 21 days of Tour de France stages.
Benjamin et al., 2020 [67]	19 NCAA DI female soccer athletes, mean age 20.6 ± 1.4 years.	GPS (100 Hz accelerometers) WBGT	Statistically significant differences in relative distance, relative high-speed running distance, and relative high metabolic load were observed with increasing WBGT risk categories for hyperthermia. Individuals differed in effects of performance associated with heat acclimation. Decreases in relative high-speed running distance seemed to negatively correlate with aerobic fitness level as well.
Bozzini et al., 2021 [64]	20 NCAA DI female beach volleyball players, mean age 20 ± 1 years.	Integrated GPS and HR monitoring technology	Average workloads were higher in practices than matches, but match workloads surpassed those of practice when pre-match warm-ups were factored in. Athletes expended over 500 calories on average during matches as well.
Costa et al., 2021 [68]	34 Portuguese high-level outfield female soccer players, mean age 20.6 ± 2.3 years.	Sleep quality HRV (HR monitor and wrist-worn accelerometer)	Sleep duration ranged between 6.5 to 8.8 h and decreased after evening training sessions. Sleep efficiency ranged between 86% and 90%. Nocturnal heart rate variability indices were normal. No differences in sleep efficiency, nocturnal HRV, and perceived ratings of wellbeing were observed over the 2-week study period. Within-match workloads accounting for two matches play per day equated to two 90 min soccer games over a weekend.
Flatt et al., 2016 [69]	12 NAIA collegiate female soccer players, mean age 22 ± 2.3 years.	HRV (chest strap Polar transmitter)	Decrease in vagal HR index (Ln rMSSD) demonstrated greater improvements on Yo-Yo Intermittent Recovery Test following 5 weeks of offseason training. HRV data gathered via smartphone showed meaningful training status information.
Flatt et al., 2017 [70]	8 NCAA DI female soccer players, mean age 20.2 ± 1.8 years.	HRV (pulse-wave finger sensor) HR monitor (chest strap Polar transmitter)	Increased training load is correlated with decreased cardiac parasympathetic modulation (a measure sensitive to fatigue). The opposite was found with a decrease in training load.
Hoshikawa et al., 2013 [71]	7 intercollegiate female middle-distance runners, mean age 19.6 ± 0.8 years.	Sheet-type nocturnal sleep monitoring sensor Normobaric hypoxia room	Increased HR, RR, and restlessness, and decreased SpO_2_ were observed during hypoxic night 1. However, physiological variables progressed toward normoxic levels within 1 week.
Kupperman et al., 2021 [65]	32 NCAA DI female collegiate soccer players, mean age 20 ± 1 years.	GPS (10 and 100 Hz sampling rate)	Total distance and player loads were twice as high during practices than games. During practice sessions, defenders displayed the highest median player loads of all positions during practices, while midfielders had the highest median player loads during games.
McKeown et al., 2016 [72]	12 Australian national-level female netball athletes, mean age 19.9 ± 0.4 years.	Linear position transducer (barbell)	Loaded countermovement jumps performed resulted in power and jump height improvements at an earlier time than the unloaded condition. Frequent monitoring of performance variables across different jump types can prove more informative to coaching practices than focusing on jump height alone.
Perrotta et al., 2019 [73]	24 Canadian national team female field hockey players, mean age 22.6 ± 3.0 years	HR monitor (POLAR Team^2^, 1000 Hz sampling rate)	Significant correlation between experienced and prescribed training loads through a 5-week final preparatory mesocycle was found. Minimal deviations (−5.4 to 7.1%) in weekly prescribed training loads were also observed. Overall, fitness levels did not significantly correlate with magnitude of deviation.
Ransdell et al., 2020 [74]	6 NCAA DI female basketball players, mean age 19.7 ± 1.5 years.	Catapult Optimeye S5 unit	Athletes’ jumps increased over the 4-year playing period. Player load per minute was also higher among guard positions than posts. Athletes experienced increased high-inertial movement analysis in games that were lost than were won as well.
Sekulic et al., 2014 [75]	57 college-aged female (*n* = 21) and male (*n* = 36) athletes, mean age 21.8 ± 2.5 years.	Computer-managed agility course	Male athletes of agility-saturated sports performed better on a reactive agility test than male athletes of non-agility sports; this instance was not observed among female athletes. Reactive and nonreactive practices were found to share 36–46% common variance. All athletes performed better during the change of direction drill than the reactive agility test.
Strauss et al., 2019 [76]	30 South African sub-elite female soccer players, mean age 22.8 ± 2.4 years.	GPS (100 Hz accelerometers) HR monitor (fix Polar transmitter belt)	Positional distinctions in distance traveled during matches were observed with midfielders (84.4 m/min) reporting the greatest. Defenders spent the greatest time in the high-intensity HR zone per minute (13.3%); forwards spent the least (9.7%). Mean HR during matches: 159 bpm (81% of HR_max_). All match variables decreased in the second half.
Tian et al., 2013 [77]	34 Chinese national team female wrestlers, mean age 23.0 ± 3.0 years	HRV (OmegaWave sport technology system, millisecond sampling)	Large deviations above and below normal HRV indices (rMSSD and SDNN) lasting >2 weeks indicated nonfunctional overreaching in athletes. Associated changes in HRV indices lasted for >3 weeks, concurring with decreased physical performance, in those experiencing nonfunctional overreaching.
Vlantes & Readdy 2017 [78]	11 NCAA DI female collegiate volleyball players, aged 18–21.9 years.	Catapult Optimeye S5 Microsensors	Setters displayed the highest mean player load, as well as the highest number of jumps of all positions in a 5–1 system. Individual differences based on position were observed for changes to player loading, percentage of high-impact player load, and jumps over 3-, 4-, or 5-set matches.

Note. bpm: beats per minute; DI: division one; GPS: global positioning system; HR: heart rate; HR_max_: max heart rate; HRV: heart rate variability; Ln rMSSD: logarithm of the root mean square of successive R-R interval differences; km: kilometers; m/min: meters per minute; NAIA: National Association of Intercollegiate Athletics; NCAA: National Collegiate Athletic Association; rMSSD: square root of the mean of the sum of the squares of differences between adjacent R-to-R intervals; RR: respiratory rate; SDNN: standard deviation of all normal R-to-R intervals; SpO_2_: oxygen saturation; WBGT: wet-bulb globe temperature; 1-RM: 1-repitition maximum.

## Data Availability

No new data were created or analyzed in this study. Data sharing is not applicable to this article.
